# Parametrization of Combined Quantum Mechanical and Molecular Mechanical Methods: Bond-Tuned Link Atoms †

**DOI:** 10.3390/molecules23061309

**Published:** 2018-05-30

**Authors:** Xin-Ping Wu, Laura Gagliardi, Donald G. Truhlar

**Affiliations:** Department of Chemistry, Chemical Theory Center, and Supercomputing Institute, University of Minnesota, Minneapolis, MN 55455-0431, USA

**Keywords:** electrostatics, molecular modeling, multiscale modeling, QM/MM

## Abstract

Combined quantum mechanical and molecular mechanical (QM/MM) methods are the most powerful available methods for high-level treatments of subsystems of very large systems. The treatment of the QM−MM boundary strongly affects the accuracy of QM/MM calculations. For QM/MM calculations having covalent bonds cut by the QM−MM boundary, it has been proposed previously to use a scheme with system-specific tuned fluorine link atoms. Here, we propose a broadly parametrized scheme where the parameters of the tuned F link atoms depend only on the type of bond being cut. In the proposed new scheme, the F link atom is tuned for systems with a certain type of cut bond at the QM−MM boundary instead of for a specific target system, and the resulting link atoms are call bond-tuned link atoms. In principle, the bond-tuned link atoms can be as convenient as the popular H link atoms, and they are especially well adapted for high-throughput and accurate QM/MM calculations. Here, we present the parameters for several kinds of cut bonds along with a set of validation calculations that confirm that the proposed bond-tuned link-atom scheme can be as accurate as the system-specific tuned F link-atom scheme.

## 1. Introduction

The combined quantum mechanical and molecular mechanical (QM/MM) method is now well established for molecular simulations of large and complex chemical systems that are too big to be treated accurately by full QM methods [1,2,3,4,5,6]. The method combines the accuracy of QM methods and the efficiency of MM methods by treating a small-scale primary system at the QM level and a large-scale secondary system at MM level. Examples include catalytic systems where one needs to include effects beyond the active site and QM/MM methods have been widely applied to both enzyme kinetics [5,6,7,8,9,10,11,12,13,14,15,16,17,18,19,20,21,22] and metal-organic frameworks (MOFs) [23,24,25,26,27,28,29,30,31].

The treatment of the QM−MM boundary is a challenging issue within the framework of QM/MM, especially when the boundary between the QM fragment and the MM surroundings passes through a covalent bond, which is unavoidable in treating enzymes and MOFs. A number of approaches have been proposed to cap the dangling bonds of the QM subsystem, such as a link atom or pseudoatom [32,33,34,35,36,37,38,39,40,41,42,43,44,45], or localized or generalized hybrid orbitals [46,47,48,49]. (Note that the H* method of Chaquin [39,41,42] was not used in the context of QM/MM calculations.) The link atom scheme is the subject of the present article.

**Figure 1:**
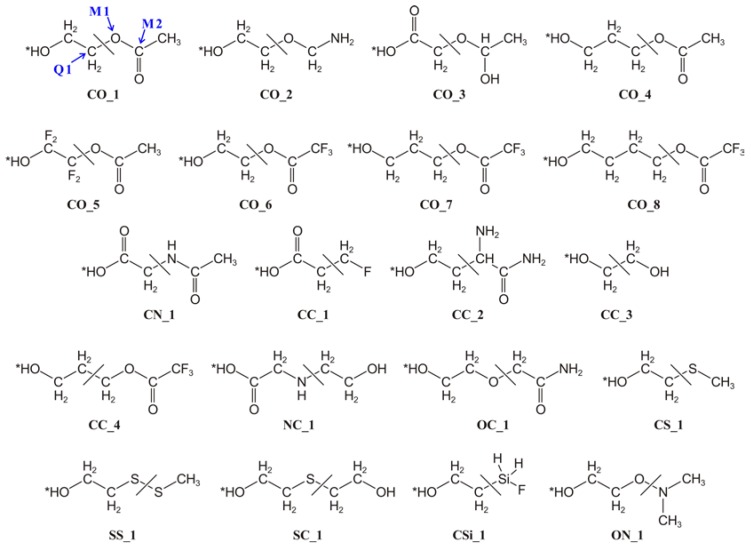
Test Suite. The asterisk (*) denotes a deprotonation site. The QM region is on the left of the cut bond, and the MM region is on its right. The Q1, M1, and M2 atoms of CO_1 are labeled. This test suite includes the whole test suite (15 molecules) that used in previous works [50,51] plus five new molecules, i.e., CO_5, CO_6, CO_7, CO_8, and CC_4.

Within the link-atom scheme, an H atom is the most widely used choice, with the QM−MM boundary usually set at C−C bonds because electrostatic balance is less of an issue at nonpolar bonds; nevertheless, a previous work [45] found that a F atom tuned for a particular structure of a particular system can perform better than H as a link atom in most cases, especially when the MM boundary atom is electronegative. The reason for choosing F as a tuned atom is that it is the most electronegative element and the preferred tuning procedure is to add a repulsive pseudopotential. Countervailing this success, the original tuned F link-atom scheme has some limitations. First, it is generally limited to QM/MM systems having just one kind of cut bond because there is no general scheme to tune F link atoms used to cap inequivalent cut bonds in the same fragment. Second, it is sometimes hard to choose a representative structure for the reaction of interest because multiple structures play an important role in the reaction. Third, the need for tuning is cumbersome for high-throughput screening. Here, we propose a new way to get the tuning parameters that overcomes all three of these drawbacks of the original scheme. The link atoms obtained by the new scheme are called *bond-tuned link atoms*. The new scheme is validated against a data set of 20 QM/MM systems involving 10 different cut bonds, as illustrated in the test set shown in Figure 1.

## 2. Method

A QM/MM calculation proceeds by dividing the entire system (ES) into two parts: a subsystem that will be treated by QM and a subsystem that will be treated by MM. In the present work, we assume that these subsystems are connected by a covalent bond. When the QM subsystem is pulled out of the ES, the bond is cut and the atom on the QM side of the bond (this atom is called Q1, see CO_1 in Figure 1 as an example) is unsaturated. A link atom is added to make a bond to Q1, and the QM subsystem combined with the link atom is called the capped QM system; the QM subsystem without the link atom is called the uncapped QM subsystem. The link atom is often taken to be a normal (nontuned) hydrogen atom, but in the present work, we take it to be a tuned fluorine atom. The definition of a tuned link atom is that it has a parameter varied so that the charge on the uncapped QM subsystem in a calculation on the capped QM system is the same as the charge on the uncapped QM subsystem in a calculation on the ES or a good model of the ES. The ES or model thereof that is used for tuning will be called the tuning system.

As previously described [45], to construct a tuned F atom (denoted as F*), the 1 *s*^2^ core of a real F atom is replaced by the sum of the CRENBL effective core potential (ECP) for F [52] plus the tuning pseudopotential U(r). The tuning potential is [45]

(1) $$U\left(r\right)=C\;\exp\left[-{\left(r/a_{0}\right)}^{2}\right]$$

where $a_{0}$ is the Bohr radius and C (which will be given in hartrees) is the tuning parameter fitted to satisfy the definition stated above.

In applications (discussed below), the calculations on the capped QM system are carried out in the presence of background charges representing the electrostatic field due to the MM subsystem; this is called electronic embedding [53]. In previous work, we found that background charges have only a small effect on the final tuning parameter [50], and so the tuning was carried out without background charges. We followed the same protocol here.

In the tuned F link atom scheme, the tuning system is the specific system to be used in a particular QM/MM application [45]. An F atom tuned in this way is called a system-specific tuned F atom.

In the present work, we derive and validate the bond-tuned link atoms. Each of these tuned link atoms is parametrized for a particular kind of cut single bond at the QM−MM boundary, for example, where the Q1 atom is C and the other atom on the MM side of the cut bond (this atom is called M1, see CO_1 in Figure 1 as an example) is O. To derive the bond-tuned parameter for a certain kind of cut bond, we use (as the tuning system) a simple but representative molecule including that kind of bond. The present article derives parameters for 10 kinds of cut bonds, those in Figure 1, and the tuning system for each of these bond types is given in Table 1. 

To evaluate the performance of the bond-tuned link atom scheme, we considered both H link atom and tuned F link atom schemes. The H and F* link atoms were placed along the axes of Q1−M1 bonds. The standard bond lengths listed in Table 2 were employed for Q1−H and Q1−F* link-atom bonds.

As described above, the tuning process requires calculating the charges on QM subsystems. We use the CM5 charge model [54], which is especially well suited for this purpose because of its good stability with respect to changing the basis set [51], that is, the tuning parameter is not strongly dependent on the basis set if using CM5 charges. In addition, CM5 charges can nicely reproduce the dipole moments of full quantum mechanical calculations [51]. A previous study [51] showed that “as compared to Mulliken charges, the CM5 charges describe the charge distributions in test molecules better, and they reproduce the dipole moments of full quantum mechanical calculations better.”

## 3. Details of the Validation Calculations

Although we turned background charges off during the tuning process, which is called mechanical embedding [53], background charges are actually considered in all of our QM/MM applications, i.e., the more realistic electronic embedding is used in the QM/MM calculations.

Electronic embedding calculations require specifying the method for treatment of boundary charges, i.e., the charges at the MM boundary. For the treatment of boundary charges in QM/MM calculations, we used the two previously recommended charge modification schemes [50], i.e., the balanced redistributed charge-2 (BRC2) scheme [45] and the balanced smeared redistributed charge (BSRC) scheme [50]. Here, we summarize these two charge modification schemes.

As the capped QM system has the same total charge as the original entire system, the charge on the entire QM/MM system (a capped QM system plus a MM subsystem) is not the same as the total charge on the original entire system. Therefore, the first step in both the BRC2 scheme and the BSRC scheme is to adjust the charge on the MM subsystem in order to conserve the total charge of the entire QM/MM system [45,50]. More specifically, the charge on the M1 atom is adjusted to make the total charge of the MM subsystem be zero [45,50].

Then, in the BRC2 scheme, the adjusted M1 charge is redistributed to all M2 atoms (i.e., the MM atoms directly bonded to M1 atoms, see CO_1 in Figure 1 as an example) evenly; in this scheme, all MM charges are point charges.

In the BSRC scheme, the adjusted M1 charge is redistributed to the midpoints of all M1−M2 bonds evenly. In this scheme, all MM charges are point charges except the redistributed charges, which are smeared as [50]

(2) $$q_{RC}^{*}=q_{RC}-q_{RC}\;\left(1+r/r_{0}\right)\;\exp\left(-2r/r_{0}\right)$$

where $q_{RC}$ is the redistributed charge, r is the distance of the charge density from the redistributed charge center, and $r_{0}$ is the smearing width of the charge density. Here, we use the previously recommended smearing width of 1 Å [50].

The additive QM/MM scheme with electronic embedding was adopted for QM/MM calculations; in this scheme the QM/MM energy of the entire system is given by [50,55]

(3) $$E\left(\mathrm{QM}/\mathrm{MM};ES\right)=E\left(QM;CQM^{**}\right)+\left[E\left(val;ES\right)-E\left(val;CQM\right)\right]\;+\left[E\left(vdW;ES\right)-E\left(vdW;CQM\right)\right]+E\left(Coul;MM^{**}\right)$$

where $E\left(QM;CQM^{**}\right)$ is the quantum mechanical energy of the capped QM system embedded in the modified electrostatic field of the MM subsystem with adjusted M1 charge, the first bracketed energy difference is the difference in MM valence (val) energy terms between the entire system and the capped QM system, the second bracketed energy difference is the difference in MM van der Waals (vdW) energy terms between the entire system and the capped QM system, and $E\left(Coul;MM^{**}\right)$ is the Coulomb (Coul) interaction energy of the MM subsystem with adjusted M1 charge. Note that the energy differences in brackets are independent of all decisions about electrostatics and charges.

To validate the robustness of the bond-tuned link atom scheme, we used the test suite that was used in previous works [50,51] plus five new and challenging molecules (i.e., CO_5, CO_6, CO_7, CO_8, and CC_4). The CO_5 and CO_6 test molecules were respectively constructed by replacing the two CH_2_ groups in the QM subsystem of CO_1 with two strong electron-withdrawing CF_2_ groups and by replacing the CH_3_ group in the MM subsystem of CO_1 with a strong electron-withdrawing CF_3_ group. The CO_7 and CO_8 test molecules have the same QM−MM boundary and MM subsystem as CO_6, but one (for CO_7) or two (for CO_8) more CH_2_ groups are included in the QM subsystem. The CC_4 and CO_7 test molecules have the same structure and they only differ in the position of QM−MM boundary (see Figure 1). All 20 test molecules are shown in Figure 1, and the deprotonation processes of these molecules are the test reactions used to evaluate the new scheme. We considered the deprotonation processes because they provide the most sensitive test of electrostatics because they involve a whole unit change in charge. If the method works well for the deprotonation processes, it should work well for other kinds of chemical processes involving less severe changes in charge distributions.

The expression for the deprotonation energy is

(4) E(deprotonation)=E(deprotonated species)−E(protonated species)

The geometries of the protonated species were optimized at the full QM level (the Cartesian coordinates for these species can be obtained from the Supplementary Materials), while the deprotonated species were constructed by just removing a proton (the one marked with an asterisk in Figure 1) from the optimized protonated species without further optimization. Thus, we are considering what may be called vertical or sudden deprotonation, not adiabatic deprotonation. The reason for this is that the purpose of the present paper is to test the treatment of electrostatics for the demanding process of protonation/deprotonation in a way where the electrostatic effect is not obscured or partially cancelled or enhanced by geometry changes.

Determination of the tuning parameters for the bond-tuned scheme was carried out using the neutral molecules of Table 1. For testing the system-specific tuned F link atom scheme [45], tuning was performed using the protonated species of the molecules in the test suite; in addition, to be consistent with the tuning process of the bond-tuned link atom scheme, CM5 charges [54] were used and background charges were not considered while tuning. As stated above, a previous paper [50] reported that the effect of background charges on the tuning parameter is small.

All QM/MM calculations were carried out in our own *QMMM 2017* program [56], which uses *Gaussian 16* [57] as the QM engine and modified *TINKER 6.3* [58] as the MM engine. All QM calculations were performed using *Gaussian 16* [57]. The M06-2X density functional [59,60] and the 6-311G** [61] basis set were used for the calculations on the capped QM system and the entire system. The MMFF94 force field [62] was used for the valence and van der Waals terms of Equation (3). For the original MM charges (i.e., MM charges before applying the charge modification schemes), M06-2X/6-311G**/CM5 charges of the protonated species were used.

A previous paper in our group [51] has already tested the sensitivity of the tuning parameters and deprotonation energies to the basis set. That paper showed that the tuning parameters have at most minor changes with the variation of basis set; for different basis sets, the deprotonation energies are very similar. Thus, we only consider one basis set in the present paper.

## 4. Results and Discussion

### 4.1. Tuning Parameters

The tuning parameters C (in hartrees), obtained as described above, are listed in Table 3. In general, the system-specific tuning parameters are similar for molecules containing the same type of cut bond and show larger differences among molecules containing different types of cut bonds. The bond-tuned parameters are in qualitative agreement with the system-specific tuning parameters, although for CO_1, CO_4, CO_6, CO_7, CO_8, CN_1, CC_4, and OC_1 we observed relatively large deviations between the two sets of tuning parameters, with the system-specific tuning parameters typically smaller than those of the corresponding bond-tuned parameters. Equation (1) shows that for a tuning potential, a smaller tuning parameter C leads to a more electronegative tuned F atom; a positive (negative) tuning parameter means that a repulsive (attractive) tuning potential is added to a F atom. It is reasonable to infer that the molecules (CO_1, CO_4, CO_6, CO_7, CO_8, CN_1, CC_4, and OC_1) that have significantly smaller system-specific tuning parameters than bond-tuned parameters show this trend because they have strong electron-withdrawing groups (carbonyl and CF_3_) in the MM subsystems. Consistent with this hypothesis, we see that CO_5 has a stronger electron-withdrawing character than CO_1 in the QM region and has 0.2 hartree increase in the system-specific tuning parameter, while CO_6, which has stronger electron-withdrawing character in the MM region, has C decreased by 0.2 hartree. It is also consistent that CO_7 and CO_8, which have the same QM−MM boundary and MM subsystem as CO_6, have system-specific tuning parameters close to the corresponding parameter for CO_6. A final consistency check reported in Table 3 is that for the molecules that have C as the Q1 atom, but with different M1 atoms (O, N, C, S, Si), the bond-tuned parameters indicate that the electronegativities of the tuned F atoms have the order of C−Si < C−C < C−S < C−N < C−O. This order matches well with the electronegativity order of the MM boundary atoms, which lends support to the physicality of the derived bond-tuned parameters.

### 4.2. CM5 Charge Analyses

**Figure 2:**
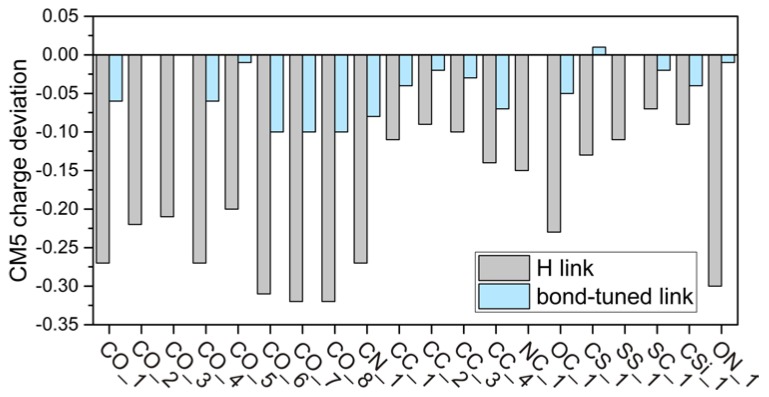
Histogram for CM5 charge deviations of the capped-QM-system results from the entire-system results shown in Table 4.

We computed the sum of the CM5 charges of the QM subsystems (i) of the entire molecules in the test suite; (ii) of the H-capped QM systems, and (iii) of the bond-tuned capped QM systems; the results are compared in Table 4. The table and Figure 2 show that the H-capped QM subsystem models fail to reproduce the total charges of the QM subsystems of the molecules, and the error can be as large as ~0.3 e. In contrast, we found that for most cases, the CM5 charges calculated from the QM subsystem capped with bond-tuned link atoms and those obtained from calculations on the entire system model are very similar, even for the molecules that have relatively large deviations of the tuning parameters (i.e., for CO_1, CO_4, CO_6, CO_7, CO_8, CN_1, CC_4, and OC_1, as discussed in Section 4.1). These results indicate that the bond-tuned link atoms perform significantly better than the H link atoms in terms of charges, and in fact, they accomplish the goal of making the charge on the QM subsystem realistic.

### 4.3. Deprotonation Energies

Table 5 shows the full QM deprotonation energies and the QM/MM signed errors and mean unsigned errors (MUEs) of the deprotonation energies (QM/MM deprotonation energies are given in the Supplementary Materials). For the QM/MM calculations, we found that for each link atom scheme, the two recommended charge modification schemes (i.e., BRC2 and BSRC) give very similar results. The key result in Table 5 is that the bond-tuned link atoms perform as well as the system-specific tuned F link atoms (MUE: 2.5 vs. 2.3 kcal/mol for the BRC2 scheme; 2.5 vs. 2.2 kcal/mol for the BSRC scheme) and much better than the H link atoms (MUE: 7.2 kcal/mol for the BRC2 scheme; 7.5 kcal/mol for the BSRC scheme). Moreover, for many molecules (e.g., OC_1), the bond-tuned link atoms even outperform the system-specific tuned F atoms in terms of QM/MM signed errors. These results show that using the bond-tuned link atom to cap the QM boundary atom can catch the main natures of the cut bond, and they indicate that the tuning parameter is transferable among molecules with the same type of cut bond. The bond-tuned link atom scheme is as straightforward and convenient as the popular H link atom scheme but as accurate as using the system-specific tuned F link atom scheme.

Table 5 also shows that the eight seemingly most challenging molecules (i.e., CO_1, CO_4, CO_6, CO_7, CO_8, CN_1, CC_4, and OC_1, as discussed above) all have similar errors between the system-specific and bond-tuned link atoms, with most (except CO_6) differences being 1.4 kcal/mol or less, though the two schemes have relatively large deviations on the tuning parameter for these molecules (see Table 3), which is very encouraging.

Compared to the system-specific tuned F link atom scheme, the existence of strong electron-withdrawing groups may introduce additional error in the bond-tuned link atom scheme. However, it is encouraging to see that the additional error is not very large (~1.2 kcal/mol in the case of CO_1, ~0.8 kcal/mol in the case of CO_4, and ~2.1 kcal/mol in the case of CO_6). In addition, the error of ~6 kcal/mol when using the bond-tuned link atom scheme in the case of CO_6 is still very small compared to the large deprotonation energy of 385.6 kcal/mol. Moreover, for CO_6, the bond-tuned link atom still performs significantly better than the H link atom. We also found that when moving the QM−MM boundary away from the reaction site (CO_6 → CO_7 → CO_8), the errors decrease significantly for all the link atom schemes. These results indicate that for QM/MM calculations employing the bond-tuned link atom scheme, increasing the size of the QM region is an effective way to reduce the error when strong electron-withdrawing (or donating) groups exist near the QM−MM boundary. We noticed that choosing the position of QM−MM boundary is also important. CC_4 and CO_7 have the same structure; they only differ in the position of QM−MM boundary, with a C−C (C−O) bond being cut for CC_4 (CO_7). Although CC_4 has a smaller QM region than CO_7, it has smaller errors than CO_7 for all the link atom schemes, which is mainly due to two reasons: (1) the strong electron-withdrawing groups are further away from the QM−MM boundary in CC_4 compared to CO_7; (2) usually setting the QM−MM boundary at a C−C bond instead of a C−O bond gives smaller errors.

## 5. Concluding Remarks

The introduction mentioned three limitations of the system-specific tuned F link-atom scheme. The bond-tuned scheme presented here overcomes all three of these limitations. It can be preparametrized with general parameters, and it can be applied to QM/MM systems including multiple cut bonds at the QM−MM boundary, for example, a MOF with the QM−MM boundary cutting through its inorganic node. A tuned F atom is just as convenient to use as the popular H link atom but is more accurate, and it has good stability [51] with respect to variation of basis sets because it is tuned with CM5 charges [54].

The overall performance (CM5 charges and deprotonation energies) of the proposed bond-tuned link-atom scheme is quite similar to the previous (system-specific) tuned F link-atom scheme [45]. The existence of strong electron-withdrawing groups near the QM−MM boundary may increase the error more when using the bond-tuned link atom scheme than when using the system-specific tuned F link-atom scheme, but the average error in the new method is still small.

Since the transferability of the tuning parameter among molecules with the same type of cut bond is validated in this work and the good stability of the tuning parameter with respect to basis set variations is validated in a previous work [51], future studies can focus on extending the database of bond-tuned parameters, which can serve for the high-throughput and accurate QM/MM calculations.

## Figures and Tables

**Table 1:** Tuning molecules for various cut bonds (the atoms or fragments appearing before and after the dashes are in the QM and MM subsystems, respectively).

**Cut bond**	C−O	C−N	C−C	N−C	O−C
**Molecule**	H_3_C−OH	H_3_C−NH_2_	H_3_C−CH_3_	H_2_N−CH_3_	HO−CH_3_
**Cut bond**	C−S	S−S	S−C	C−Si	O−N
**Molecule**	H_3_C−SH	HS−SH	HS−CH_3_	H_3_C−SiH_3_	HO−NH_2_

**Table 2:** Standard bond lengths (Å) [45].

**Bond**	C−H	N−H	O−H	S−H
**Distance**	1.09	1.01	0.95	1.34
**Bond**	C−F*	N−F*	O−F*	S−F*
**Distance**	1.33	1.41	1.41	1.65

**Table 3:** System-specific and bond-tuned parameters C (in hartrees) of the tuned F link atoms.

	System-Specific	Bond-Tuned
CO_1	−0.0185	0.2256
CO_2	0.2075	0.2256
CO_3	0.2434	0.2256
CO_4	−0.0310	0.2256
CO_5	0.1954	0.2256
CO_6	−0.2124	0.2256
CO_7	−0.2283	0.2256
CO_8	−0.2392	0.2256
CN_1	−0.0122	0.3181
CC_1	0.6806	0.8463
CC_2	0.7715	0.8463
CC_3	0.7315	0.8463
CC_4	0.5499	0.8463
NC_1	1.0798	1.0966
OC_1	0.8599	1.0625
CS_1	0.6079	0.5888
SS_1	0.8256	0.8108
SC_1	0.9750	1.0658
CSi_1	0.7520	0.9001
ON_1	0.5766	0.6321

**Table 4:** Sum of partial atomic charges of all QM atoms using the CM5 charge model and deviations of the capped-QM-system results from the entire-system results.

	Entire System	Capped QM Subsystem		Deviations	
		H Link	Bond-Tuned Link	H Link	Bond-Tuned Link
CO_1	0.185	−0.083	0.130	−0.27	−0.06
CO_2	0.131	−0.085	0.127	−0.22	0.00
CO_3	0.107	−0.103	0.111	−0.21	0.00
CO_4	0.189	−0.084	0.131	−0.27	−0.06
CO_5	0.078	−0.122	0.071	−0.20	−0.01
CO_6	0.229	−0.082	0.131	−0.31	−0.10
CO_7	0.233	−0.083	0.132	−0.32	−0.10
CO_8	0.238	−0.080	0.135	−0.32	−0.10
CN_1	0.157	−0.112	0.081	−0.27	−0.08
CC_1	−0.003	−0.112	−0.043	−0.11	−0.04
CC_2	0.003	−0.085	−0.014	−0.09	−0.02
CC_3	0.000	−0.102	−0.027	−0.10	−0.03
CC_4	0.054	−0.085	−0.015	−0.14	−0.07
NC_1	−0.154	−0.304	−0.158	−0.15	0.00
OC_1	−0.109	−0.342	−0.157	−0.23	−0.05
CS_1	0.041	−0.084	0.046	−0.13	0.01
SS_1	−0.009	−0.120	−0.006	−0.11	0.00
SC_1	−0.049	−0.120	−0.073	−0.07	−0.02
CSi_1	0.007	−0.086	−0.028	−0.09	−0.04
ON_1	−0.041	−0.340	−0.055	−0.30	−0.01
average				−0.20	−0.04

**Table 5:** QM deprotonation energies (DE), QM/MM signed errors and mean unsigned errors (MUEs) of deprotonation energies (all energies and errors in kcal/mol) for the test suite using the BRC2 and BSRC schemes with H link atoms and with system-specific and bond-tuned link atoms.

	Molecule ^1^	DE	H Link		System-Specific Tuned F Link		Bond-Tuned Link	
			BRC2	BSRC	BRC2	BSRC	BRC2	BSRC
CO_1	**HOCH_2_CH_2_**−OC(O)CH_3_	393.7	11.4	11.6	2.9	3.1	4.1	4.3
CO_2	**HOCH_2_CH_2_**−OCH_2_NH_2_	399.6	8.4	9.4	0.6	1.7	0.7	1.8
CO_3	**HOOCCH_2_**−OCHOHCH_3_	365.8	5.0	6.6	−1.0	0.6	−1.0	0.5
CO_4	**HOCH_2_CH_2_CH_2_**−OC(O)CH_3_	396.8	7.3	7.4	1.6	1.8	2.4	2.6
CO_5	**HOCF_2_CF_2_**−OC(O)CH_3_	356.7	6.3	7.2	−0.1	0.9	0.1	1.1
CO_6	**HOCH_2_CH_2_**−OC(O)CF_3_	385.6	12.5	12.4	4.0	3.8	6.1	5.9
CO_7	**HOCH_2_CH_2_CH_2_**−OC(O)CF_3_	391.0	8.2	8.1	2.4	2.2	3.7	3.6
CO_8	**HOCH_2_CH_2_CH_2_CH_2_**−OC(O)CF_3_	393.4	6.1	6.0	1.4	1.2	2.4	2.2
CN_1	**HOOCCH_2_**−NHCOCH_3_	354.7	9.0	9.4	−1.1	−0.5	0.6	1.1
CC_1	**HOOCCH_2_**−CH_2_F	360.8	4.6	4.7	−1.9	−1.8	−0.9	−0.9
CC_2	**HOCH_2_CH_2_**−CHNH_2_CONH_2_	404.2	6.2	6.2	0.3	0.3	0.8	0.7
CC_3	**HOCH_2_**−CH_2_OH	400.8	14.7	14.8	1.8	2.0	2.9	3.0
CC_4	**HOCH_2_CH_2_**−CH_2_OC(O)CF_3_	391.0	5.7	5.6	−0.9	−1.0	0.6	0.6
NC_1	**HOOCCH_2_NH**−CH_2_CH_2_OH	375.3	3.3	4.2	−3.4	−3.0	−3.3	−2.9
OC_1	**HOCH_2_CH_2_O**−CH_2_CONH_2_	397.8	3.6	4.0	−7.1	−7.0	−5.7	−5.6
CS_1	**HOCH_2_CH_2_**−SCH_3_	393.7	12.8	13.4	6.4	7.0	6.3	6.9
SS_1	**HOCH_2_CH_2_S**−SCH_3_	388.6	3.9	4.2	1.0	1.4	1.0	1.3
SC_1	**HOCH_2_CH_2_S**−CH_2_CH_2_OH	392.7	−0.8	−0.4	−2.4	−2.2	−1.9	−1.7
CSi_1	**HOCH_2_CH_2_**−SiH_2_F	394.5	7.7	6.4	1.6	0.2	2.4	1.1
ON_1	**HOCH_2_CH_2_O**−N(CH_3_)_2_	398.6	6.3	8.3	−4.0	−2.3	−3.6	−1.9
MUE			7.2	7.5	2.3	2.2	2.5	2.5

^1^ The QM subsystem is in bold font in the table.

## Supplementary Materials

The following are available online at http://www.mdpi.com/1420-3049/23/6/1309/s1. QM/MM deprotonation energies and Cartesian coordinates for molecules (optimized using M06-2X/6-311G**) in the test suite.
