# Supplementary material for: Parametrization of Combined Quantum Mechanical and Molecular Mechanical Methods: Bond-Tuned Link Atoms
Source: Molecules. 2018 May 30;23(6):1309. doi: 10.3390/molecules23061309 (PMC6100187; doi:10.3390/molecules23061309)
Supplement: Supplementary file 1 [file molecules-23-01309-s001.pdf]

SUPPLEMENTARY MATERIALS

May 20, 2018

Parametrization of Combined Quantum Mechanical and Molecular Mechanical Methods: Bond-Tuned Link Atoms

Xin-Ping Wu, Laura Gagliardi, and Donald G. Truhlar

Department of Chemistry, Chemical Theory Center, and Minnesota Supercomputing Institute, University of Minnesota, Minneapolis, Minnesota 55455-0431, United States.

CONTENTS

1. Corresponding Authors S-2
2. QM/MM Deprotonation Energies S-3
3. Cartesian Coordinates for Molecules (Optimized Using M06-2X/6-311G**) in the Test Suite S-5

1. CORRESPONDING AUTHORS

Xin-Ping Wu:

ORCID: 0000-0003-3147-8333

Email: xwuphd@umn.edu

Laura Gagliardi:

ORCID: 0000-0001-5227-1396

Email: gagliard@umn.edu

Donald G. Truhlar:

ORCID: 0000-0002-7742-7294

Email: truhlar@umn.edu

2. QM/MM DEPROTONATION ENERGIES

Table S1. QM/MM deprotonation energies (in kcal/mol) for the test suite using the BRC2 and BSRC schemes with H link atoms and with system-specific and bond-tuned link atoms

	H link		system-specific tuned F link		bond-tuned link	
molecule	BRC2	BSRC	BRC2	BSRC	BRC2	BSRC
CO_1	405.0712550	405.2631381	396.5574807	396.8285595	397.7629467	398.0257807
CO_2	408.0696850	408.9873243	400.2557930	401.3387303	400.3549374	401.4358040
CO_3	370.8192671	372.3837596	364.8176137	366.3840728	364.7678315	366.3360163
CO_4	404.0638721	404.2043959	398.4128818	398.5812591	399.2290762	399.3922603
CO_5	362.9543305	363.8937681	356.5799627	357.5746759	356.7349539	357.7253196
CO_6	398.0982040	397.9807641	389.5588206	389.3408009	391.6499167	391.4442447
CO_7	399.1964718	399.0581259	393.3453539	393.1519242	394.7135076	394.5309387
CO_8	399.4712927	399.3537926	394.7312225	394.5777405	395.7497571	395.6047535
CN_1	363.7135251	364.0974357	353.5680739	354.1530975	355.3078938	355.8046024
CC_1	365.4434392	365.5043819	358.9136108	358.9661397	359.8513266	359.9007912
CC_2	410.4500969	410.3820924	404.5654590	404.4971234	404.9982495	404.9305996
CC_3	415.5223112	415.6738250	402.6931199	402.8179829	403.7203386	403.8378482
CC_4	396.6454045	396.6114206	390.0559698	390.0076155	391.6222218	391.5768426
NC_1	378.6249360	379.4652557	371.8632636	372.3044472	371.9760659	372.4153466
OC_1	401.3257534	401.7571963	390.6296129	390.7692851	392.0477139	392.1815482
CS_1	406.4192662	407.0089096	400.0586256	400.6805365	399.9553531	400.5773083
SS_1	392.5145982	392.8638184	389.6680176	390.0469208	389.5861770	389.9650248
SC_1	391.9685909	392.3064427	390.3774301	390.5791021	390.8698637	391.0635180
CSi_1	402.1272038	400.8149707	396.0537930	394.7064232	396.8653026	395.5218562
ON_1	404.9688497	406.9221882	394.5990146	396.3133290	394.9979346	396.7048401

Table S2. Calculated QM and QM/MM energies (in a.u.) for the test molecules in the test suite

molecule	QM	QM/MM					
		H link		system-specific tuned F link		bond-tuned link	
		BRC2	BSRC	BRC2	BSRC	BRC2	BSRC
CO_1	-382.862832	-155.007009	-155.009791	-178.717737	-178.727689	-177.974024	-177.984266
CO_2	-324.853295	-154.988655	-154.999360	-177.995165	-178.032697	-177.940875	-177.978490
CO_3	-458.085398	-229.059819	-229.071580	-251.952667	-251.994553	-252.005921	-252.047716
CO_4	-422.167031	-194.311234	-194.313591	-218.060900	-218.069295	-217.278516	-217.287168
CO_5	-779.855079	-551.993815	-552.005979	-575.064469	-575.102426	-574.973864	-575.011944
CO_6	-680.582195	-154.972287	-154.969771	-179.294964	-179.286554	-177.944869	-177.936030
CO_7	-719.886341	-194.276347	-194.273396	-218.649617	-218.639611	-217.249189	-217.238646
CO_8	-759.191135	-233.579671	-233.576964	-257.989375	-257.979401	-256.554289	-256.543737
CN_1	-437.037761	-229.070824	-229.097254	-252.750427	-252.842585	-251.750052	-251.844504
CC_1	-367.585496	-229.047064	-229.048983	-250.666355	-250.673682	-250.200003	-250.207435
CC_2	-418.349932	-155.009696	-155.008618	-176.379606	-176.374951	-176.170360	-176.165663
CC_3	-230.216356	-115.688672	-115.693346	-137.178924	-137.197531	-136.856904	-136.875701
CC_4	-719.886341	-154.964243	-154.962762	-176.971998	-176.967126	-176.129243	-176.124247
NC_1	-438.207058	-284.393160	-284.408001	-304.882576	-304.951099	-304.838032	-304.906649
OC_1	-438.192677	-230.240797	-230.251484	-251.295686	-251.346597	-250.751095	-250.802844
CS_1	-592.489851	-154.995906	-154.998452	-176.830362	-176.838855	-176.885210	-176.893673
SS_1	-990.676988	-553.196114	-553.199476	-574.419748	-574.429677	-574.460369	-574.470271
SC_1	-707.002672	-553.176255	-553.190942	-573.992862	-574.051094	-573.750060	-573.808738
CSi_1	-544.987098	-155.000808	-154.993397	-176.428832	-176.399014	-176.015142	-175.984575
ON_1	-364.105744	-230.130797	-230.151728	-251.952275	-252.038300	-251.796878	-251.883495

3. CARTESIAN COORDINATES FOR MOLECULES (OPTIMIZED USING M06-2X/6-311G**) IN THE TEST SUITE

****

CO_1

****

C	-1.97338800	-0.58123300	-0.00051600
H	-1.93037200	-1.22170500	0.88816600
H	-1.93058000	-1.21979000	-0.89058400
C	-0.78214700	0.34952800	0.00036700
H	-0.78733700	0.98962100	-0.88363300
H	-0.78746600	0.98794600	0.88559000
O	0.38193300	-0.48146800	-0.00034700
C	1.56020000	0.17027400	-0.00057300
C	2.71059300	-0.79874000	0.00033500
H	2.65180000	-1.43260300	0.88588100
H	2.64364400	-1.44544500	-0.87517300
H	3.64569000	-0.24577000	-0.00747500
O	1.64798400	1.36470600	-0.00020300
O	-3.11729600	0.24875700	0.00051200
H	-3.89789400	-0.30718800	-0.00014300

****

CO_2

****

C	1.74712500	-0.53321200	-0.06266100
H	1.69438300	-1.05417000	-1.02525800
H	1.65751000	-1.27584100	0.73857600
C	0.58631600	0.43158500	0.03590300
H	0.63999500	0.97023700	0.99234300
H	0.65720600	1.17132200	-0.77366200
O	-0.59784000	-0.31938500	-0.06284700
C	-1.74898600	0.48767700	-0.01882300
H	-1.67950000	1.14051800	0.86973600
H	-1.79519200	1.13045900	-0.90859000
N	-2.89200000	-0.38635000	-0.02785400
H	-3.02333500	-0.81566000	0.88002300
H	-3.73329400	0.12235500	-0.26611400
O	2.92759700	0.23796200	0.05923900
H	3.68144500	-0.34968700	-0.00971600

****

CO_3

****

C	0.56309900	-0.66489400	0.19315400
H	0.53725200	-1.63575300	-0.31066600
H	0.44618800	-0.84340600	1.27337100
C	1.95760100	-0.10578700	0.00440100
O	2.93361700	-0.79716400	-0.06531100
O	-0.40613300	0.21957800	-0.28545100
C	-1.68459500	0.07134600	0.31188200
H	-1.53617500	0.04142700	1.40555800
C	-2.40240300	-1.16922600	-0.17018700
H	-1.86204900	-2.07267100	0.11809200
H	-3.40325500	-1.20676500	0.26002100
H	-2.47984700	-1.12273500	-1.25711000
O	-2.43543300	1.17418900	-0.06414700
H	-1.89586500	1.95415800	0.09825700
O	1.99173700	1.23080300	-0.02278000
H	2.92124100	1.47786300	-0.12151200

****

CO_4

****

C	-2.67966000	0.43166400	0.00142000
H	-2.68838300	1.08027500	-0.88495500
H	-2.68768900	1.07601300	0.89086000
C	-1.42648900	-0.42329700	-0.00105500
H	-1.43521500	-1.06926500	0.87974300
H	-1.43729800	-1.06698400	-0.88351500
C	-0.17359800	0.42573600	-0.00136200
H	-0.11669800	1.06982600	-0.88317100
H	-0.11602300	1.06941000	0.88071900
O	0.94869500	-0.46465000	-0.00205500
C	2.15638100	0.12728400	-0.00071000
C	3.25796200	-0.89702400	0.00167300
H	3.16966800	-1.52318700	0.89025500
H	3.15628800	-1.54410700	-0.87013300
H	4.21927200	-0.39103300	-0.01063100
O	2.30476400	1.31699700	0.00032900
O	-3.78848000	-0.44778200	-0.00044700
H	-4.59133000	0.07635800	0.00841000

****

CO_5

****

C	1.54936100	0.31254800	-0.00003300
F	1.59487500	1.12170100	1.07960300
F	1.59524900	1.12070700	-1.08040300
C	0.21323100	-0.44745500	0.00009200
F	0.15782600	-1.22668500	-1.08460500
F	0.15740100	-1.22558600	1.08557600
O	-0.78853600	0.48846800	-0.00055900
C	-2.10717000	0.04835200	-0.00043100
C	-3.01938600	1.23511700	0.00004700
H	-2.82170700	1.84200300	0.88437900
H	-2.81448400	1.84929300	-0.87748000
H	-4.04973800	0.89123800	-0.00517600
O	-2.40241200	-1.09931800	-0.00016500
O	2.55687200	-0.57528700	0.00053900
H	3.39414800	-0.09602900	0.00017800

****

CO_6

****

C	-2.82864600	-0.64666200	-0.00080600
H	-2.69123500	-1.27310000	0.88783800
H	-2.69135200	-1.27024200	-0.89147700
C	-1.79163200	0.45308800	0.00085800
H	-1.88380800	1.08253200	-0.88530900
H	-1.88394600	1.07995000	0.88885200
O	-0.50830600	-0.19606800	0.00003900
C	0.53993100	0.61091100	-0.00061300
C	1.83097700	-0.22642800	-0.00015000
F	1.88190400	-1.00110600	1.08394800
F	1.87551100	-1.01411100	-1.07492100
F	2.89248200	0.56251500	-0.00804500
O	0.53051300	1.80137700	-0.00079600
O	-4.07588400	0.01391800	0.00033500
H	-4.77309600	-0.64408300	-0.00109800

****

CO_7 and CC_4

****

C	-3.73128300	0.31937600	0.00000500
H	-3.82343100	0.95914400	-0.88811800
H	-3.82342100	0.95916600	0.88811300
C	-2.37785100	-0.36817300	0.00000200
H	-2.30413000	-1.00784500	0.88202500
H	-2.30412100	-1.00782000	-0.88203800
C	-1.24832500	0.63629500	0.00001400
H	-1.26434300	1.27830000	-0.88424500
H	-1.26426400	1.27820700	0.88434200
O	-0.01259400	-0.10601500	-0.00008000
C	1.08848100	0.62485300	-0.00008500
C	2.31930500	-0.29916500	-0.00000100
F	2.31414100	-1.08082400	1.07991500
F	2.31372900	-1.08168400	-1.07930500
F	3.43247700	0.41578600	-0.00049700
O	1.16309200	1.81421100	-0.00002000
O	-4.71091300	-0.69923100	0.00002800
H	-5.57807200	-0.28948500	-0.00012600

****

CO_8

****

C	-3.20261900	0.34266100	0.00060700
H	-3.30217100	0.98663600	-0.87839600
H	-3.30268300	0.98430900	0.88125100
C	-1.84688400	-0.35780900	0.00009800
H	-1.74953900	-1.00056000	0.87998800
H	-1.74930400	-0.99885200	-0.88101600
C	-0.71461700	0.64398600	0.00118200
H	-0.73253600	1.28545400	-0.88303300
H	-0.73301800	1.28403800	0.88642600
C	-4.35526600	-0.64304500	-0.00100600
H	-4.28940000	-1.28711200	0.88629200
H	-4.28919500	-1.28445700	-0.89020400
O	0.52185500	-0.10055200	0.00097200
C	1.62536000	0.62598300	-0.00030800
C	2.85212300	-0.30348900	-0.00016200
F	2.84482900	-1.08371400	1.08132600
F	2.84153100	-1.08893500	-1.07779200
F	3.96908500	0.40525200	-0.00356200
O	1.70614100	1.81482400	-0.00122400
O	-5.55348200	0.10892800	-0.00001300
H	-6.29585000	-0.49820600	-0.00139200

****
CN_1
****

C	0.58002200	-0.60237100	0.00112600
H	0.55075600	-1.25630200	0.88121000
H	0.55088600	-1.25976000	-0.87633500
C	1.87584700	0.18530000	0.00001000
O	2.99510300	-0.55761400	0.00096500
O	1.92585400	1.37759900	-0.00137600
N	-0.52876700	0.30911500	-0.00113900
H	-0.31284800	1.29508000	-0.00131500
C	-1.80055900	-0.17344600	-0.00181000
C	-2.90104500	0.86339500	0.00149700
H	-3.49658500	0.72806200	0.90475500
H	-2.53035400	1.88726800	-0.03833100
H	-3.54977200	0.67658700	-0.85419600
O	-2.02336700	-1.36688400	-0.00140600
H	2.78298200	-1.49681800	0.00177800

****
CC_1
****

C	-0.23444600	-0.58605800	-0.00032000
H	-0.31061500	-1.23166500	0.88012500
H	-0.31073800	-1.23067100	-0.88148500
C	1.10936100	0.11199900	-0.00014000
O	1.23948000	1.29783700	-0.00011200
C	-1.36867900	0.41896000	0.00028200
H	-1.33132300	1.04952200	0.88878700
H	-1.33140000	1.05044600	-0.88756700
F	-2.56529800	-0.27565300	-0.00001000
O	2.18767900	-0.69842300	0.00022900
H	1.91706900	-1.62145900	0.00036100

****
CC_2
****

C	-0.48071400	0.61758900	0.33886300
H	-0.36969400	0.61644000	1.43033700
C	0.91381300	0.62009800	-0.29445300

H	0.83172100	0.47556900	-1.37661800
N	-1.29461300	1.78946800	-0.00348000
H	-0.92390000	2.61250200	0.45759900
H	-1.22785600	1.96492200	-1.00266100
C	-1.25268900	-0.67213800	0.02575000
N	-2.59139000	-0.51173300	-0.07245100
H	-2.98473700	0.40878400	0.04233100
H	-3.16481500	-1.32425700	-0.22531400
O	-0.69262000	-1.74361000	-0.09146700
H	1.35080300	1.61080800	-0.13176800
C	1.89590800	-0.39153500	0.27513000
H	1.64429400	-1.39658900	-0.06392300
H	1.83979300	-0.37510600	1.37330200
O	3.18612800	0.00854500	-0.16757800
H	3.80044000	-0.70078400	0.02884700

****

CC_3

****

C	-0.00571400	-0.00992400	-0.75605300
C	0.00571400	0.00992400	0.75605300
H	1.04666400	0.03521700	1.09978500
H	-0.49282700	0.92411300	1.09971800
H	-1.04666400	-0.03521700	-1.09978500
H	0.49282700	-0.92411300	-1.09971800
O	0.66793500	1.15681800	-1.18659900
H	0.67381500	1.16721900	-2.14490300
O	-0.66793500	-1.15681800	1.18659900
H	-0.67381500	-1.16721900	2.14490300

****

NC_1

****

C	2.53227900	0.46643700	0.20853700
H	2.32969300	1.42323000	-0.28671600
H	2.55895700	0.64104000	1.29360800
C	1.41751000	-0.50699000	-0.12163400
H	1.65460200	-1.48260100	0.32320700
H	1.37926000	-0.63852400	-1.20560400
O	3.73220700	-0.12344200	-0.24617700
N	0.13248000	0.02593800	0.32622000
H	0.16051300	0.15785400	1.33368700

C	-1.01400000	-0.81126900	-0.01074700
H	-1.17160900	-1.66934200	0.64965300
H	-0.87261100	-1.20072500	-1.02329600
C	-2.30403100	0.00908200	-0.04317200
O	-2.10250200	1.32188500	-0.18680800
O	-3.38918900	-0.48460900	0.02145900
H	4.45501800	0.48577600	-0.08581100
H	-1.13585500	1.44748700	-0.18795800

****
OC_1
****

C	-2.54724800	0.51318400	-0.03214700
H	-2.44823700	1.09675400	-0.95419800
H	-2.44355900	1.19249800	0.82119300
C	-1.43443300	-0.50985200	0.02054800
H	-1.51934900	-1.09866000	0.94410400
H	-1.52553100	-1.19658400	-0.83219300
O	-0.21471500	0.19194400	-0.02175200
O	-3.76097900	-0.21333800	0.00991300
C	2.16707800	0.17699900	0.00468400
O	2.18094000	1.37962700	0.02413400
N	3.29968700	-0.58684700	-0.02169400
H	3.27659800	-1.58921500	-0.08884500
H	4.18854300	-0.11543800	-0.05158700
C	0.88743800	-0.65463900	0.02657800
H	0.88494700	-1.26440300	0.94349800
H	0.89795100	-1.34744500	-0.82942500
H	-4.48813500	0.41040700	-0.01703500

****
CS_1
****

C	1.59516000	-0.42605000	0.00017000
H	1.60501100	-1.06846200	-0.88877000
H	1.60499600	-1.06784100	0.88955300
C	0.33786300	0.42267900	-0.00012000
H	0.33243700	1.05722000	0.88829500
H	0.33265000	1.05691700	-0.88875300
S	-1.13226000	-0.64536400	-0.00009300
C	-2.39553400	0.65248700	0.00013500
H	-2.31247400	1.27216200	-0.89279700

H	-3.36612600	0.15884800	0.00028200
H	-2.31218700	1.27214400	0.89305400
O	2.68836500	0.47344800	-0.00015200
H	3.50000100	-0.03744000	0.00072400

****
SS_1
****

C	-1.00874500	0.21573000	-0.27724800
H	-1.00053800	0.17197800	-1.36561700
H	-0.79501000	1.23576300	0.04363600
S	1.95166400	-0.19479700	-0.57430500
C	2.42136800	1.16558900	0.53573200
H	2.61614100	0.78660300	1.53679300
H	3.33353300	1.59974500	0.12450500
H	1.64451200	1.92794700	0.56527800
S	0.26408000	-0.91772700	0.37602900
C	-2.37018100	-0.20101000	0.24960700
H	-2.60115300	-1.21731300	-0.09174700
H	-2.36074000	-0.19886700	1.34675700
O	-3.29617700	0.74011900	-0.25917100
H	-4.17388900	0.49171800	0.03763100

****
SC_1
****

C	1.37327000	-0.41384800	0.00029300
H	1.31183500	-1.04425700	-0.88835500
H	1.31199200	-1.04387100	0.88922600
S	0.00000000	0.77733500	0.00028800
C	-1.37327100	-0.41384700	0.00074500
H	-1.31142900	-1.04498600	-0.88735700
H	-1.31240000	-1.04313900	0.89022200
C	2.69740800	0.32631500	0.00000100
H	2.76209100	0.96459200	0.88952100
H	2.76150600	0.96493900	-0.88932100
C	-2.69740800	0.32631500	-0.00074000
H	-2.76220200	0.96584700	0.88787600
H	-2.76139400	0.96368400	-0.89096400
O	-3.70929900	-0.66377300	0.00001000
H	-4.56197200	-0.22470300	-0.00177100
O	3.70929900	-0.66377300	-0.00055000

H	4.56197300	-0.22470100	-0.00116000

****

CSi_1

****

C	1.61509400	0.43673900	-0.00028700
H	1.60328900	1.08419400	0.88692500
H	1.60312300	1.08323600	-0.88818900
C	0.39390600	-0.46803300	0.00029900
H	0.43825600	-1.11869900	-0.87822300
H	0.43856700	-1.11794000	0.87936500
Si	-1.22698800	0.45244200	0.00015700
H	-1.36997900	1.28632400	-1.21101300
H	-1.37053300	1.28582900	1.21159700
F	-2.42949300	-0.63006100	-0.00032500
O	2.76304000	-0.39324200	0.00006700
H	3.54222700	0.16710800	-0.00034500

****

ON_1

****

C	2.06948800	0.00082300	0.49946800
H	2.03855900	-0.88731300	1.14159400
H	2.03834900	0.89064400	1.13924500
C	0.85530900	-0.00061900	-0.40448600
H	0.86245200	0.88738500	-1.04514100
H	0.86318200	-0.89024300	-1.04290200
O	-0.27782600	0.00009800	0.44136300
N	-1.45895900	-0.00007600	-0.35390600
C	-2.18928800	-1.20277500	0.01646200
H	-2.42398500	-1.22466500	1.08970800
H	-3.11500100	-1.23602100	-0.56037700
H	-1.58694200	-2.07504100	-0.23677500
C	-2.18920800	1.20284600	0.01585900
H	-3.11476900	1.23603000	-0.56122800
H	-2.42420000	1.22516700	1.08904000
H	-1.58663100	2.07491200	-0.23749000
O	3.20218100	-0.00010600	-0.34891600
H	3.98904600	-0.00191800	0.19826500
